# Flexible school attendance strategies for epidemic outbreaks: simulation-based decision support

**DOI:** 10.1186/s12889-025-25197-4

**Published:** 2025-12-02

**Authors:** Satoshi Takahashi, Masaki Kitazawa, Atsushi Yoshikawa

**Affiliations:** 1 Graduate School of Economics, Nagoya University, Chikusaku, Nagoya, Aichi 4648601 Japan; 2 Graduate School of Management, GLOBIS University, Nibancho, Chiyoda, Tokyo 1020084 Japan; 3 Kitazawa Tech, Yokohama, Kanagawa Japan; 4 College of Science and Engineering, Kanto Gakuin University, Kanazawa, Yokohama, Kanagawa 2368501 Japan

**Keywords:** Emerging infectious diseases, School environment, UNESCO policies, Timetable adjustments, Infectious disease simulation, Social continuity, Educational continuity

## Abstract

**Background:**

Emerging infectious diseases require time before appropriate countermeasures can be established because of the uncertainty surrounding their infection characteristics. During the COVID-19 pandemic, this delay led to significant disruptions in both the education sector and society as a whole. However, it is challenging to impose uniformly strict infection control measures in educational settings because the importance of continuity of education and social stability must also be considered.

**Methods:**

Characteristics of emerging infectious diseases, such as latent period, infectious period, transmission potential, and asymptomatic rate, gradually become clearer over time. This study therefore proposed a framework to evaluate effective infection control measures at different stages (early, middle, and late) of an emerging infectious disease, as more information becomes available. This framework enables decision-making about educational opportunities and social continuity to be adjusted to reflect the characteristics of the disease known at any given time. The value of this framework was verified through simulations. It was used to compare the effectiveness of four staggered school attendance strategies recommended by UNESCO, under various disease characteristics (latent period, infectious period, transmission potential, and asymptomatic rate).

**Results:**

The most effective intervention varies with the characteristics of the infectious disease (latent period, infectious period, transmission potential, and asymptomatic rate), demonstrating the importance of our framework. This framework makes it possible to select appropriate school strategies based on the known characteristics of the disease at different stages of an outbreak, when considering the school environment and economic conditions.

**Conclusions:**

This study proposed a framework for the phased adjustment of infection control measures in schools with increasing knowledge of the characteristics of an emerging infectious disease. Simulations demonstrated the effectiveness of the framework. This framework enables the selection of effective staggered school attendance methods that balance infection suppression with educational and social considerations. The framework could be extended to other environments, such as workplaces and public facilities, providing a comprehensive infection control strategy that mitigates social and educational burdens. Future research should explore the framework’s application to a broader range of infectious disease scenarios to enhance its feasibility for real-world policy implementation.

## Background

In recent years, globalization has accelerated the spread of pathogens across borders, via increased international travel, trade, and urbanization. This has amplified the global threat posed by emerging infectious diseases [1]. The emergence of COVID-19 is a good example of this. First reported in Wuhan, China in late 2019, by March 11, 2020, COVID-19 had been reported in 114 countries, leading the WHO to declare a pandemic [2]. Between January 2020 and December 2021, the World Health Organization estimated that approximately 14.9 million excess deaths were associated with the COVID-19 pandemic (range 13.3–16.6 million) [3]. The pandemic caused unprecedented disruption to healthcare systems and economies worldwide [4].

When an emerging infectious disease first appears, its transmission characteristics are unclear, making it difficult to establish appropriate infection control measures. At the onset of the COVID-19 pandemic, countries adopted different responses of varying effectiveness [5]. COVID-19 also had a high proportion of asymptomatic carriers, who unknowingly contributed to the spread of the virus, further accelerating the outbreak [6]. Eventually, widespread vaccination programs were established. However, before that occurred, measures such as movement restrictions were found to be effective, leading to their global adoption [7].

The COVID-19 pandemic also highlighted the risks associated with the global spread of infectious diseases [8].

In particular, there was a higher risk of infection spreading in densely populated areas, necessitating strong cooperation and surveillance measures around the world [9]. The economic impact of the COVID-19 outbreak was severe, with travel restrictions and stagnation in economic activities affecting society as a whole [10].

Given the threat posed by emerging infectious diseases, particularly in light of the COVID-19 pandemic, conventional infection control measures in schools have been reevaluated to prepare for future outbreaks. Students interact in close proximity within school environments, and specialized measures are therefore required to mitigate infection risks. Proposed countermeasures range from staggered attendance schedules to long-term school closures [11, 12].

These strategies may vary in their effectiveness in curbing infection spread and their impact on education and society. For instance, UNESCO has proposed four staggered school attendance strategies (UNESCO1, UNESCO2A, UNESCO2B, and UNESCO3) as infection control measures for schools. UNESCO states that these strategies progressively enhance infection control but also increase negative effects on education and society [13]. For example, extending home learning periods is effective in reducing infection spread. However, it deprives students of educational opportunities and places additional burdens on school staff and parents, leading to significant economic consequences. School staff must provide learning materials for students staying at home, and parents must oversee their children’s studies and meals at home. Educational continuity and social stability must therefore be carefully considered when deciding on infection control measures, making it challenging to implement uniformly strict measures in schools. For instance, closing schools each time a new infectious disease emerged would significantly disrupt both education systems and society.

The characteristics of emerging infectious diseases are largely unknown upon discovery but gradually become clearer over time. In the early stages of an emerging infectious disease, characteristics such as the latent period and infectious period are identified through contact tracing and secondary infection analysis [14–16]. In the middle stage, transmission potential, including the basic reproduction number, is assessed using animal studies and statistical investigations to determine how easily the disease spreads [17, 18]. In the late stage, large-scale testing and antibody surveys help to clarify the proportion of asymptomatic carriers and their role in transmission [19–23]. Appendix A shows how the characteristics of SARS and COVID-19 were identified over time.

Selecting school attendance strategies that align with the stage-specific characteristics of an emerging infectious disease, while balancing infection control, education continuity, and social stability, is considered an effective approach. This paper therefore proposes a framework that uses infection simulations to inform the selection of appropriate school strategies at each stage (early, middle, and late) as the characteristics of an emerging infectious disease become clearer.

The structure of this paper is as follows: Methods section describes the proposed framework and the methodology used for its evaluation. Results section presents the verification results and shows the importance and effectiveness of the proposed framework. Finally, Discussion section provides conclusions and discusses directions for future research.

## Methods

### Definitions

In this study, several epidemiological terms are used. Exposed limit days refers to the latent period, i.e., the time from exposure to becoming infectious. Infecting exposed limit days denotes the infectious period, i.e., the time during which an individual can transmit the disease. Quantum generation rate is the rate at which infectious particles are emitted by an infected person via airborne transmission. Asymptomatic rate is the proportion of infected individuals who do not show symptoms but may still spread the infection.

### Proposal for a framework

**Fig. 1:**
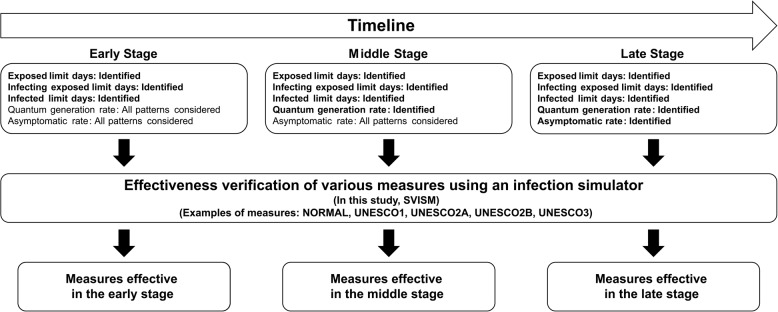
Proposed framework

We proposed a framework for flexible adjustments to school attendance policies that respond to the stage at which the characteristics of an infectious disease are identified, while also considering the impact on education and society. The overall structure of the proposed framework is shown in Fig. 1.

The proposed framework categorizes the progression of an infectious disease into three stages: early, middle, and late stage. These stages correspond to the identification of specific disease characteristics: - Early stage: The exposed limit days and infecting exposed limit days are determined. - Middle stage: The quantum generation rate is identified. - Late stage: The asymptomatic rate is known.

At each stage, the framework drew on the characteristics identified by that point to conduct simulations of infection spread. For characteristics that were unknown at that stage, multiple scenarios were considered based on realistic assumptions about their possible values and combinations. The simulation evaluated the effectiveness of various school attendance strategies (e.g., UNESCO’s proposed staggered attendance policies) in mitigating infection spread under these conditions.

The results for each stage were then used to determine the most effective strategies based on the known characteristics of the infectious disease at that time. By assessing the impact of multiple school attendance strategies at different stages, the framework enables dynamic and flexible decision-making that considers factors such as school operations, educational continuity, economic stability, and household conditions.

Note that the terms early stage, middle stage, and late stage do not indicate that a single simulation run progresses sequentially through these stages. Instead, they represent analytical viewpoints corresponding to different points in time after the emergence of a novel infectious disease, when different subsets of epidemiological characteristics have been identified. For each stage, only the parameters that would realistically be known at that time were treated as fixed, while the remaining parameters were considered as unknowns, and varied across plausible values. All simulations were conducted in advance for the full factorial combination of parameter values, and results were later aggregated by the set of parameters known at each stage. This design allowed us to evaluate which intervention strategies would be most effective given the level of epidemiological knowledge available at that stage.

### Verifying the effectiveness of the framework

To verify the effectiveness of the proposed framework, two types of evaluations were conducted.

**Fig. 2:**
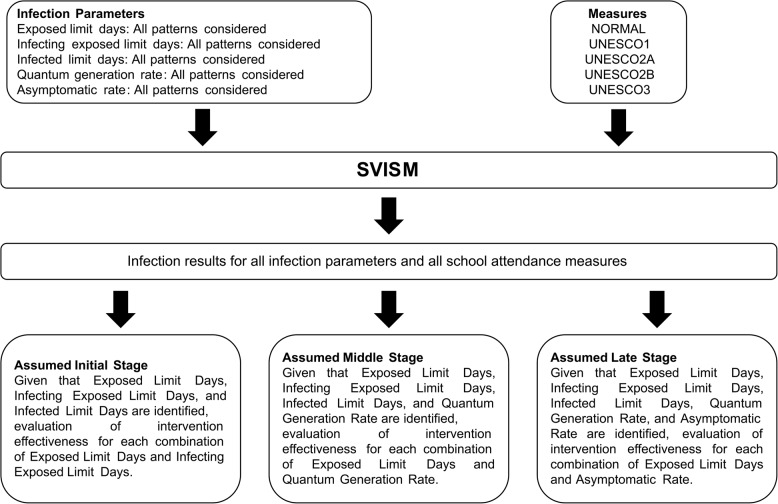
Experiment design

The first evaluation assessed the overall effectiveness of various school attendance strategies against different infectious diseases. It examined whether the optimal strategy varied by the stage at which disease characteristics were identified (early, middle, or late stage) and the nature of these characteristics. This evaluation demonstrated the importance of the proposed framework. The methodology for this evaluation is outlined in Fig. 2.

In this evaluation, simulations were conducted for various combinations of infectious disease parameters and school attendance strategies. The results were then analyzed at each of the three stages (early, middle, and late). Importantly, rather than running separate simulations for each stage, a comprehensive set of simulations was conducted in advance for all possible combinations of disease characteristics and school attendance strategies. The results were then aggregated and analyzed differently depending on the stage, to see whether the most effective strategy varied with the disease characteristics known at that stage.

The second evaluation applied the proposed framework to specific infectious diseases. The objective was to determine whether the framework could be used to select appropriate school attendance strategies while considering factors such as school operations, learning outcomes, economic conditions, and household environments. This evaluation provided evidence of the framework’s effectiveness. Two types of epidemiological scenarios were explored in this study: (1) a COVID-19 scenario, based on parameter values derived from published estimates of SARS-CoV-2 transmission dynamics, and (2) a hypothetical pathogen scenario, designed to test the model’s applicability under a wider range of epidemiological conditions. Both scenarios were evaluated using the same simulation framework described below.

The following sections describe the simulation model used for validation, the parameters used in the simulations, the staggered attendance schedules, and the criteria for determining the effectiveness of each school attendance strategy.

#### Simulation model

We used the School Virus Infection Simulation Model (SVISM) [24, 25] to simulate disease transmission within school settings. SVISM was specifically designed to simulate the spread of viral infections within schools. It accounts for classroom layouts, student behavior patterns, and the transmission mechanisms of the disease. SVISM is based on an extended Susceptible-Exposed-Infectious-Removed model and uses an agent-based model approach [26–33].

**Fig. 3:**
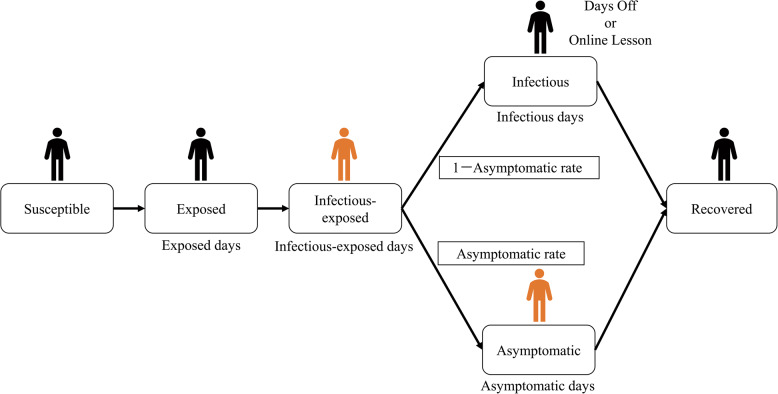
Infection model (reproduced from [24]). It includes the states susceptible, exposed, infectious-exposed, infectious, asymptomatic, and recovered

In SVISM, student states are classified as Susceptible, Exposed, Infectious-Exposed, Infectious, Asymptomatic, and Recovered (see Fig. 3). Students in the Exposed state have no risk of infecting others. Those in the Infectious-Exposed state can transmit the virus. Susceptible students can become Exposed based on a calculated infection probability, and Exposed students become Infectious-Exposed after a certain period. Infectious-Exposed individuals probabilistically develop into either Infectious or Asymptomatic cases. Infectious and Asymptomatic individuals recover after a specified period. Infectious-Exposed and Asymptomatic individuals are capable of spreading the infection to Susceptible students based on infection probability. Infectious students show symptoms and either take a leave of absence or attend classes online. Asymptomatic students continue attending school as usual.

The infection probability is calculated on the basis of an extended version of the Wells–Riley equation proposed by Dai [34–36]:

1 $$\begin{aligned} P = \frac{C}{S} = 1-e^{-Iqpt(1-n_{I})(1-n_{S})/Q} \end{aligned}$$

where *P* is the infection probability, *C* the number of new infection cases, *S* the number of Susceptible individuals, *I* the number of infectors (including Infectious-Exposed and Asymptomatic individuals), *q* is the quantum generation rate, *p* is the pulmonary ventilation rate of students, *t* is the duration of class intervals, $n_{I}$ and $n_{S}$ are the filtration efficiencies for inhalation and exhalation, and *Q* is the ventilation rate of the classroom with clean air.

**Fig. 4:**
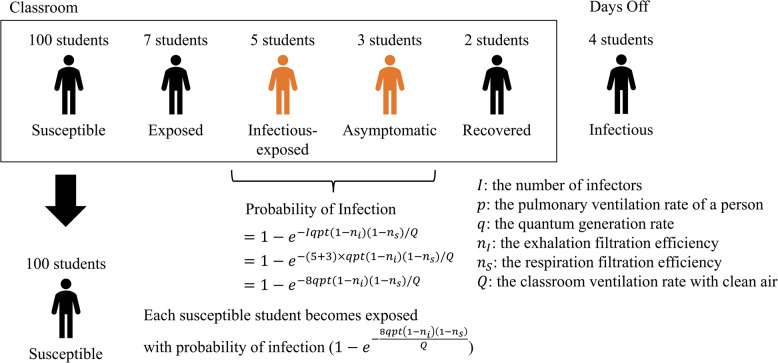
Example calculation for infection (reproduced from [24]). In this example, 100 susceptible students, five infectious-exposed students, and three asymptomatic students are present in the same classroom

Each student attends classes based on their schedule, with the infection probability during each class calculated using Equation (1). Figure 4 shows an example of new infection case calculations. The Wells–Riley equation is generally used to calculate the basic reproduction number of an infection (*C*/*I*). The number of new infection cases is determined by multiplying the number of Susceptible individuals by Equation (1). However, the number of students in classrooms often falls below 100 and the infection probability is typically below 0.01. This results in fewer than one new infection cases. We therefore used Equation (1) as the infection probability for each Susceptible individual. The expected value for new infection cases therefore aligns with the basic reproduction number over multiple simulations. In the example shown in Fig. 4, 100 Susceptible students, five Infectious-Exposed students, and three Asymptomatic students share a classroom. Here, the value of *I* in Equation (1) is 8, and each Susceptible student has an exposure probability of $(1-e^{-8qpt(1-n_{I})(1-n_{S})/Q})$.

#### Parameter settings

Table 1 shows the settings for school parameters. The school parameters were set in line with Takahashi et al. [24]. In this setup, 480 students are divided among 20 classrooms where classes are held. Each student attends different classes (classrooms) and interacts with different students. Each day consists of 7 hours, and classes continue over 12 weeks. The classroom volume (150 m$^3$) reflects the average size of a Japanese lower secondary school classroom, which is generally 60–65 m$^2$ in floor area with a ceiling height of 3 m [37]. The air change rate was set to three times per hour (3 ACH), which falls within the recommended range of 2.5–5 ACH for school classrooms [38]. The corresponding ventilation rate (450 m$^3$/h) was calculated as the product of classroom volume and air change rate. The duration of each class (50 minutes) is specified in the School Education Act Enforcement Regulations [39]. The maximum number of classes per day (7) and the number of weeks per semester (12) were assumed in this study, because they are not explicitly prescribed by law. These values were chosen to reflect both regulatory standards and typical educational practices in Japan, ensuring realistic simulation conditions. These parameter values are presented as a representative case study. However, the primary aim of this study was to demonstrate and evaluate the proposed simulation framework rather than to provide policy-specific recommendations. In this scenario, one student is randomly selected on the first day to be in the Exposed state, meaning the term begins with a single student infected outside school. We also assumed all students wear masks, with exhalation and inhalation filter efficiencies set to 0.5 [34]. “Departmentalized classrooms” describes an arrangement in which students move between specialized classrooms for different subjects (e.g., science, art), and do not simply remain in a single homeroom. This arrangement alters contact patterns and ventilation exposure in the model [24].

**Table 1:** Departmentalized classrooms parameters

Item	Value
Classroom volume	150 m^3^
Classroom air change rate	Three times/h
Classroom ventilation rate with clean air ^*a*^ (*Q*)	450 m^3^/h
Lesson time (*t*)	50 min
Total number of students	480 students
Number of classrooms per lesson	20 classrooms
Lesson weeks ^*b*^	12 weeks
Number of lessons per day	7 lessons
Pulmonary ventilation rate of a person, *p*	0.54 m^3^/h

“Departmentalized classrooms” describes an arrangement in which students move between specialized classrooms for different subjects (e.g., science, art), and do not simply remain in a single homeroom. This arrangement alters contact patterns and ventilation exposure in the model [24]

^*a*^Classroom volume multiplied by classroom air change rate

^*b*^Each week includes 5 lesson days and 2 days off

Table 2 shows the settings for infection parameters. In our framework, all infection-related parameters were treated as fixed categorical values within each simulation scenario, rather than as averages of probability distributions. For example, when *Exposed limit days* was set to 5 and *Infecting exposed limit days* to 9, all agents followed these values throughout the latent and infectious periods. Here, *Exposed limit days* corresponded to the latent period (time from exposure to becoming infectious), and *Infecting exposed limit days* corresponded to the infectious period (time during which transmission was possible). This deterministic setting enabled systematic comparisons across scenarios while eliminating stochastic fluctuations that could obscure the relative effectiveness of school attendance strategies.

**Table 2:** Infection parameters with mathematical symbols

Item	Value
Exposed limit days	1, 4, 7, 10, 13, 16 days
Infecting exposed limit days	1, 4, 7, 10, 13, 16 days
Infected limit days (*I*)	4, 7, 10, 13, 16, 19 days
Quantum generation rate (*q*)	1, 32, 64, 128, 256, 512, 1024, 2048, 4096 quanta/h
Asymptomatic rate	0.00, 0.25, 0.50
Infected asymptomatic limit days	2, 4, 6, 8, 10, 11 days (60% of infected limit days)
Exhalation filter efficiency ($n_{I}$)	0.5
Inhalation filter efficiency ($n_{S}$)	0.5

The quantum generation rate (q, unit: quanta/h) is the rate at which infectious quanta are emitted by an infected individual via airborne transmission, as defined in the Wells–Riley model [35]. Reported values of q vary widely across pathogens. For example, measles has been estimated at 60–5580 quanta/h in school and hospital outbreaks [40]; tuberculosis at 13 quanta/h depending on patient and environmental conditions [41]; rhinovirus at 1–10 quanta/h [42]; 66.91 quanta/h based on data from an elementary school setting [43]; and SARS (severe acute respiratory syndrome) has been estimated with a geometric mean of 28.77 quanta/h from a hospital outbreak [43]. These studies demonstrate that pathogen-specific infectiousness can span several orders of magnitude, from less than 10 quanta/h (e.g., rhinovirus) to several thousand quanta/h (e.g., measles).

To reflect this wide range, we adopted values for q on a logarithmic scale (1, 32, 64, 128, 256, 512, 1024, 2048, and 4096 quanta/h), rather than on a linear scale (e.g., 1, 10, 100, 1000 quanta/h). Using logarithmic spacing enables the simulation framework to capture both low- and high-infectivity pathogens within a manageable number of parameter levels, while ensuring that no range of values is overrepresented. In other words, this approach balances comprehensive coverage of the plausible epidemiological space with computational feasibility, allowing the framework to represent diverse airborne disease scenarios rather than being limited to COVID-19.

The ranges for the latent period, infectious period, and symptomatic period were determined with reference to epidemiological studies of COVID-19, SARS and other related airborne diseases [14, 15, 17]. The latent period was set to 2–14 days (median around 5 days), the infectious period to 7–14 days, and the symptomatic period to 7–21 days. These ranges reflect both short-term scenarios (e.g., mild COVID-19 cases with a latent period of 2–4 days and an infectious period within 7 days) and long-term scenarios (e.g., SARS cases with a latent period exceeding 10 days and symptomatic periods lasting approximately 3 weeks). COVID-19 data therefore provide the primary empirical basis, but the parameters were generalized to capture broader features of coronavirus outbreaks. Public health guidelines [44] were also consulted to align the parameter settings with real-world policy considerations such as isolation periods and school attendance restrictions.

The asymptomatic rate was set at 0.0, 0.25, and 0.50 to reflect the uncertainty in the proportion of asymptomatic infections reported for COVID-19 and similar diseases [19, 45]. These three levels allowed us to model scenarios ranging from all symptomatic to half asymptomatic.

Various patterns are conceivable for the symptomatic period of asymptomatic individuals. However, to prevent combinatorial explosion and consider realistic scenarios, we calculated the asymptomatic period based on the infectious period of COVID-19 and related findings. The Centers for Disease Control and Prevention estimates an infection period of approximately 14 days for COVID-19 [44]. Bullard et al. found that Vero cells infected with SARS-CoV-2 were only infectious for 8 days after symptom onset. They therefore estimated an asymptomatic period of 8 days [46]. From this, we used the formula 14 (symptomatic period) $\times$ 0.6 = 8 (asymptomatic period) and calculated the asymptomatic period by multiplying each infectious period by 60%.

#### Class schedule

**Fig. 5:**
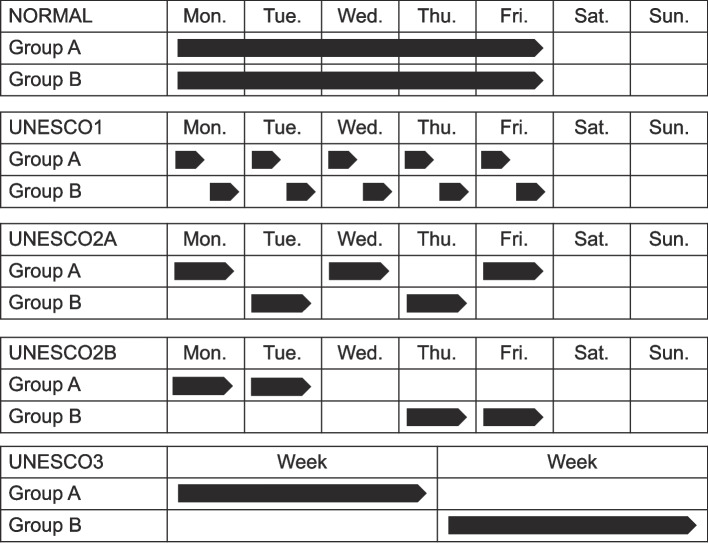
UNESCO staggered attendance policies (UNESCO1, UNESCO2A, UNESCO2B, UNESCO3) (reproduced from [24])

The class schedule used UNESCO’s four attendance patterns [13] (Fig. 5). These schedules use different attendance patterns across hours, days, or weeks, to reduce infection risk.

The details of each are as follows:

*UNESCO1*: All students attend school every day, but are split between morning and afternoon sessions.

- *Advantages*: Students attend daily, reducing learning and well-being risks. Students can ask teachers directly about online content, and regular interactions with classmates enhance emotional connections.
- *Disadvantages*: Parents may face challenges working, and coordinating schedules can be difficult. Face-to-face time is limited. Managing the schedule is complex for teachers with multiple classes, and cleaning is needed between morning and afternoon sessions.

*UNESCO2A*: Students attend school every other day in alternating shifts.

- *Advantages*: Students have regular classes with familiar teachers, minimizing confusion. While in school, they follow a standard daily schedule and can clarify any questions after the previous day’s remote learning.
- *Disadvantages*: Students do not attend school daily, which poses learning and well-being risks. Alternative childcare may be required, and scheduling can be difficult for parents and schools. Students may be affected by frequent schedule changes.

*UNESCO2B*: Students attend school for 2 or 3 consecutive days in alternating shifts.

- *Advantages*: Students benefit from consecutive days with teachers, and deep cleaning is only required once a week. This schedule provides stability for students and school operations, with remote learning days available for teacher planning and training.
- *Disadvantages*: Students are away from school for longer at a time than with UNESCO2A.

*UNESCO3*: Different grade levels attend school on a weekly basis in rotation.

- *Advantages*: Students attend a full week of standard classes and are exposed to all subjects. The school operates on a regular daily schedule.
- *Disadvantages*: Students experience extended periods away from school, and teacher face-to-face time is not maximized.

UNESCO states that infection risk decreases progressively across these schedules [13]. The week-based UNESCO3 is expected to have the lowest infection risk, and the hour-based UNESCO1 the highest.

Each schedule has both advantages and disadvantages, and it is essential to select the most appropriate one based on the situation. For example, UNESCO1, where students attend on a consistent weekly schedule, provides operational stability for schools and regular peer interactions that can foster emotional bonds. However, it can present scheduling challenges for parents.

By contrast, schedules like UNESCO2A, where students attend on alternate days, reduce contact risk but provide regular face-to-face time with teachers. However, managing the schedule may be complex for teachers handling multiple grades or subjects, potentially increasing the burden of coordination. Attendance under this schedule is adjusted each week to ensure balanced attendance days across groups.

UNESCO2B’s format, where students attend school for 2 consecutive days followed by remote learning, facilitates concentrated learning periods. However, it may be difficult for working parents to manage the frequent changes of routine required.

UNESCO3, with alternating weekly attendance, effectively reduces infection risk. However, prolonged remote learning may present challenges in maintaining both academic progress and student motivation.

Note that the disadvantages described in this section, such as reduced learning opportunities and increased administrative workload, were not incorporated as compensatory payoffs in the simulation model. The framework in this study focuses solely on epidemiological outcomes (infection dynamics) as the evaluation criterion.

#### Evaluation of intervention effectiveness based on peak infection count

We evaluated the effectiveness of interventions against infectious diseases with various characteristics based on the peak infection count ($I_{\text {peak}}$). The peak infection count is defined as the maximum number of simultaneous infections at the peak of an outbreak and is a crucial indicator of the burden on medical institutions. The ability of healthcare facilities and public services to manage limited resources during an outbreak is a critical factor in controlling the spread of infection [47]. In the early stages of a pandemic, limitations in hospital bed availability and intensive care capacity may arise, raising concerns about whether the healthcare system can handle the situation. The peak infection count ($I_{\text {peak}}$) is therefore an appropriate metric for evaluating the effectiveness of interventions to reduce the burden on healthcare systems.

The effectiveness of each intervention was evaluated based on the average peak infection count ($\text {mean } I_{\text {peak}}$) obtained from 100 simulation runs for each intervention strategy (NORMAL, UNESCO1, UNESCO2A, UNESCO2B, UNESCO3) across different infectious disease characteristics. NORMAL was the standard schedule without any staggered attendance measures.

The effectiveness of each intervention was classified into five categories based on its necessity and effectiveness: - (I) No Additional Measures Needed - (II) Very Strong Mitigation Effect - (III) Strong Mitigation Effect - (IV) Moderate Mitigation Effect - (V) No Significant Effect

To determine these classifications, two threshold levels were set: - Very Strong Mitigation Threshold: average peak infection count is no more than 10% of the total student population (48 students). - Strong Mitigation Threshold: average peak infection count is no more than 20% of the total student population (96 students).

**Fig. 6:**
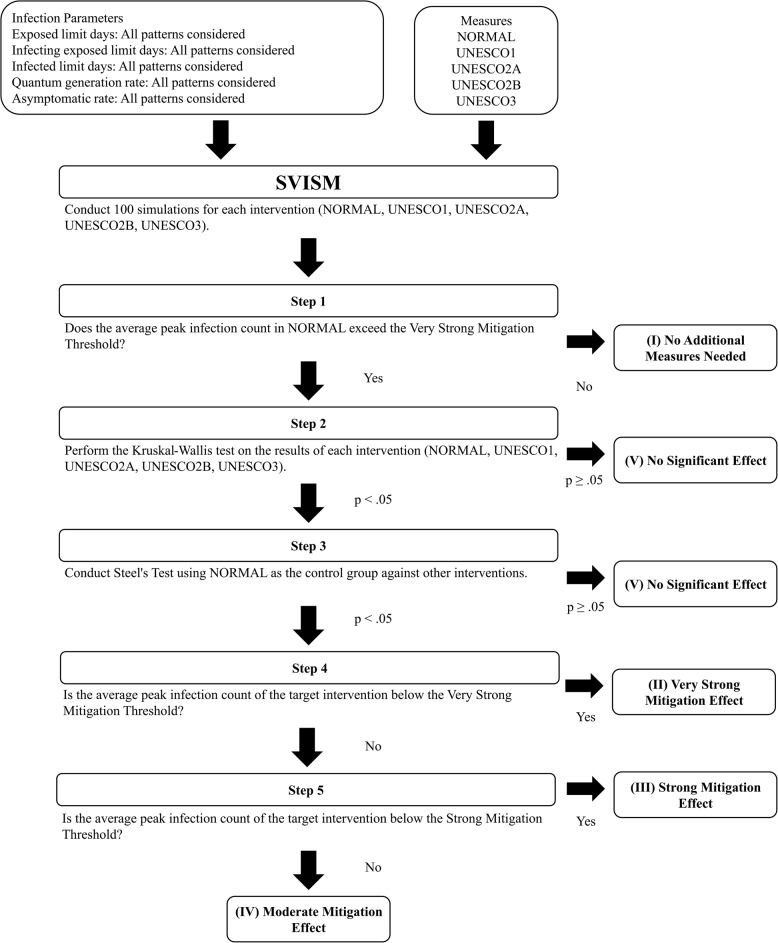
Evaluation procedure

These values can be adjusted to reflect policy decisions. The evaluation procedure is illustrated in Fig. 6.

We evaluated the effectiveness of interventions against infectious diseases with various characteristics based on the *peak infection count* ($I_{\textrm{peak}}$). Here, $I_{\textrm{peak}}$ is defined as the maximum number of simultaneous symptomatic infections observed on any single day during an outbreak. For each of the 100 simulation runs per intervention strategy, we first identified this maximum daily number of infected individuals. In cases where multiple peaks occurred during the simulation period, only the largest peak was considered. The average of these 100 peak values (*mean* $I_{\textrm{peak}}$) was then used for the statistical evaluation procedure shown in Fig. 6.

This approach was adopted because the highest peak shows the point of greatest potential strain on the healthcare system, which is a critical metric for public health capacity planning. The primary goal of many non-pharmaceutical interventions is to reduce this peak demand to a level that can be managed by available resources, such as hospital beds and intensive care units. This is often known as “flattening the curve”. This logic is consistent with previous influential modeling studies, including Ferguson et al. [48], Anderson et al. [49], and Prem et al. [50], all of which emphasize peak demand as a key indicator for evaluating intervention effectiveness. In this context, reducing the maximum $I_{\textrm{peak}}$ is a crucial indicator of whether an intervention can prevent the healthcare system from being overwhelmed.

- *Step 1: Does NORMAL exceed the Very Strong Mitigation Threshold?*
  Step 1 determines whether staggered attendance measures are necessary. If the average peak infection count under NORMAL does not exceed the Very Strong Mitigation Threshold, staggered attendance measures are not needed, and the intervention is classified as “(I) No Additional Measures Needed”.
- *Step 2: Are there differences among intervention strategies?*
  Step 2 assesses whether staggered attendance measures are effective compared to NORMAL. A Kruskal–Wallis test is conducted on the results for each intervention strategy (NORMAL, UNESCO1, UNESCO2A, UNESCO2B, UNESCO3). - If $p \ge 0.05$, there are no significant differences among the interventions, and the classification is “(V) No Significant Effect”. - If $p < 0.05$, proceed to Step 3.
- *Step 3: Are there significant differences from NORMAL?*
  In Step 3, Steel’s Test is conducted using NORMAL as the control group, with Holm’s multiple comparison adjustment applied to $p$-values. - If $p \ge 0.05$, no significant differences are found between any of the intervention strategies and NORMAL, and the classification is “(V) No Significant Effect”. - If $p < 0.05$, proceed to Step 4.
- *Step 4: Can the average peak infection count be reduced below the Very Strong Mitigation Threshold?*
  If the average peak infection count falls below the Very Strong Mitigation Threshold, the intervention is deemed highly effective and classified as “(II) Very Strong Mitigation Effect”.
- *Step 5: Can the average peak infection count be reduced below the Strong Mitigation Threshold?*
  If the average peak infection count falls below the Strong Mitigation Threshold, the intervention is classified as “(III) Strong Mitigation Effect”. If the average peak infection count exceeds the Strong Mitigation Threshold but remains lower than NORMAL, the intervention is classified as “(IV) Moderate Mitigation Effect”.

In this Section, the classification procedure was adopted to summarize the effectiveness of each intervention across the large number of parameter combinations tested. This facilitates a concise comparison and interpretation in policy-relevant terms. The raw 

$I_{\textrm{peak}}$ values contain additional information such as variance within parameter combinations, but the large number of simulation runs (100 per combination) and combinations themselves made direct visualization less interpretable. Therefore, the complete distributions of $I_{\textrm{peak}}$ values for each parameter set are not presented in this paper.

In our proposed method, the distributional shape was not taken into account. This is because the statistical tests we used (the Kruskal-–Wallis test and Steel’s test with Holm’s multiple comparison adjustment) are non-parametric methods that compare central tendency under the assumption of similar distribution shapes across groups. Steel’s test, like the Mann–Whitney U test on which it is based, assesses differences in medians when shapes are similar. However, when distribution shapes differ, the test statistic instead reflects stochastic dominance—the probability that a randomly chosen value from an intervention group is lower than one from the control group—rather than a strict median difference. We verified that our main conclusions remained robust under both interpretations.

## Results

This section provides the results of two evaluations. The first evaluation assessed the overall effectiveness of intervention measures for various infectious diseases. It examined whether the effectiveness of measures varies with the stage at which the characteristics of the infectious disease are revealed (early, middle, and late stages) and the specific characteristics themselves. This evaluation demonstrates the need for the proposed framework by showing that fixed, one-size-fits-all school attendance policies may be suboptimal. Instead, adapting policies dynamically in response to evolving epidemiological knowledge can significantly improve both infection control and educational continuity.

The second evaluation applied the proposed framework to a specific infectious disease. The objective was to verify whether the framework can help select appropriate measures while considering school operations, learning outcomes, regional economies, and household environments.

In all simulation runs, the initial stage for each classroom was set to Susceptible for all students, except for the index cases introduced in line with the parameter settings described in Section 2.3.2. The index cases were randomly assigned to classrooms at the start of each simulation, ensuring that the initial distribution followed the same conditions across all intervention strategies.

### Example of results

**Fig. 7:**
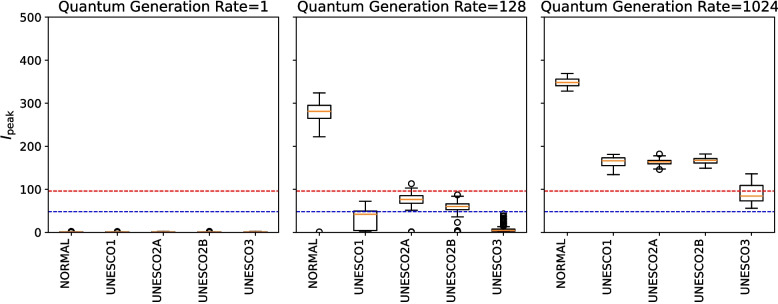
Example of simulation results under different quantum generation rates (1, 128, 1024) with exposed limit days = 7, infecting exposed limit days = 1, infected limit days = 10, and asymptomatic rate = 0.25. The blue dashed line represents the very strong mitigation threshold, defined as 10% of the total student population (48 students). The red dashed line represents the Strong Mitigation Threshold, defined as 20% of the total student population (96 students)

Figure 7 shows the distribution of $I_{\textrm{peak}}$ under the conditions of exposed limit days = 7, infecting exposed limit days = 1, infected limit days = 10, quantum generation rate = 1, 128, and 1024, and asymptomatic rate = 0.25. This illustrates how the output distribution changes when only the parameter of the quantum generation rate is increased.

First, when the quantum generation rate was 1, the distribution of $I_{\textrm{peak}}$ in the NORMAL scenario fell below the Very Strong Mitigation Threshold, and all measures were classified as “(I) No Additional Measures Needed.” This indicates that, under this parameter, no additional interventions would be required.

Next, when the quantum generation rate was 128, the distribution of $I_{\textrm{peak}}$ in the NORMAL scenario exceeded the Strong Mitigation Threshold, suggesting that some intervention was necessary. The distributions for UNESCO1 and UNESCO3 largely fell below the Very Strong Mitigation Threshold and were therefore classified as “(II) Very Strong Mitigation Effect”. However, the distributions for UNESCO2A and UNESCO2B fell between the Strong and Very Strong Mitigation Thresholds, and were classified as “(III) Strong Mitigation Effect”. This result implies that UNESCO1 and UNESCO3 are effective measures.

Finally, when the quantum generation rate was 1024, the distribution of $I_{\textrm{peak}}$ in the NORMAL scenario rose further, again indicating that some intervention would be necessary. The distributions of UNESCO1, UNESCO2A, and UNESCO2B exceeded the Strong Mitigation Threshold, but their values were significantly lower than for NORMAL. They were therefore classified as “(IV) Moderate Mitigation Effect”. The distribution for UNESCO3, however, fell between the Strong and Very Strong Mitigation Thresholds, and it was therefore classified as “(III)Strong Mitigation Effect”. This result implies that only UNESCO3 remained effective under this scenario. However, school-based measures alone were insufficient in this scenario, and the implementation of additional interventions would be desirable.

### Evaluation of the broad applicability of the framework

The visualization of results was conducted sequentially for the early, middle, and late stages, using the gradual increase in knowledge of the characteristics of infectious diseases. Visualizing all intervention measures and considering every possible combination of infectious disease characteristics would cause a combinatorial explosion of parameters. The evaluation therefore focused on intervention measures based on the characteristics identified in the early stage, particularly the exposed limit days, which is a crucial parameter of infectious diseases. It also added any newly revealed characteristics.

For the intervention analysis, the percentage of scenarios in which each intervention was effective under different parameter sets was visualized. For example, Supplementary Fig. 18 in Appendix B shows the evaluation results for different combinations of exposed limit days and infecting exposed limit days.

In Supplementary Fig. 18 in Appendix B, the vertical axis represents the combinations of exposed limit days and infecting exposed limit days. The horizontal axis represents the effectiveness classification for each intervention measure (UNESCO1, UNESCO2A, UNESCO2B, UNESCO3) in five categories: “(I) No Additional Measures Needed”, “(II) At Least Very Strong Mitigation Effect”, “(III) At Least Strong Mitigation Effect” and “(IV) At Least Moderate Mitigation Effect”.

The values in each cell are the percentage of scenarios in which each intervention was classified into each effectiveness category for a given parameter set. For example, the bottom-left cell indicates that when exposed limit days was 16 and infecting exposed limit days was 7, the percentage of scenarios classified as “(I) No Additional Measures Needed” was 11.11%. There are six levels of infected limit days (4, 7, 10, 13, 16, and 19), nine levels of quantum generation rate (1, 32, 64, 128, 256, 512, 1024, 2048, and 4096), and three levels of asymptomatic rate (0.0, 0.25, and 0.50). Each cell therefore represents the percentage of “(I) No Additional Measures Needed” scenarios among 162 possible parameter combinations.

To facilitate comparisons among intervention measures, the proportions were accumulated from “(I) No Additional Measures Needed” up to“(IV) Moderate Mitigation Effect”, and then labeled as “at least” that level. For example, the percentage of scenarios classified as “(III) At Least Strong Mitigation Effect” includes all scenarios classified as “(I) No Additional Measures Needed”, “(II) Very Strong Mitigation Effect”, or “(III) Strong Mitigation Effect”.

In reality, the six levels of infected limit days, the nine levels of quantum generation rate, and the three levels of asymptomatic rate do not occur with equal probability. However, because their actual probability distributions are unknown, we assumed an equal probability distribution and based our intervention evaluation on the percentage of effective scenarios.

The purpose of the overall analysis was to examine whether the effectiveness of intervention measures varies with the stage at which the characteristics of an infectious disease are revealed (early, middle, and late stages) and the specific characteristics themselves. The analysis therefore focused only on overall trends in intervention effectiveness across parameter combinations, without attempting to disentangle the specific epidemiological mechanisms—such as contact rate reductions, timing of peak suppression, or interaction effects between interventions—that produced these patterns.

### Early stage

**Fig. 8:**
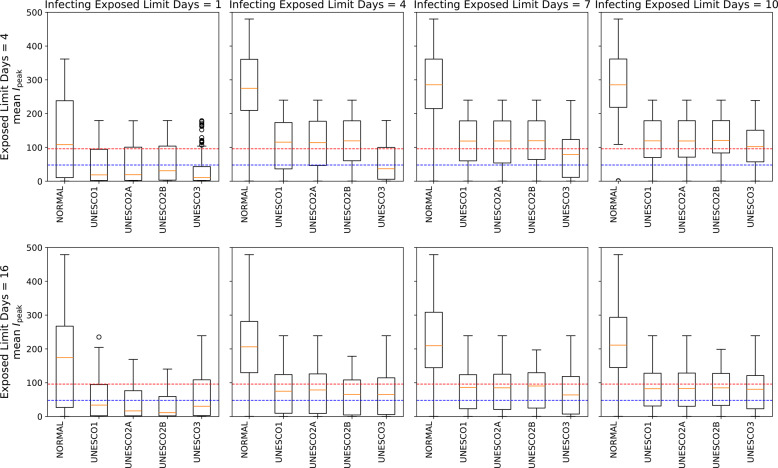
Effectiveness of UNESCO attendance strategies in the early stage (exposed limit days = 4 or 16; infecting exposed limit days = 1, 4, 7, and 10). The blue dashed line represents the very strong mitigation threshold, defined as 10% of the total student population (48 students). The red dashed line represents the strong mitigation threshold, defined as 20% of the total student population (96 students)

**Fig. 9:**
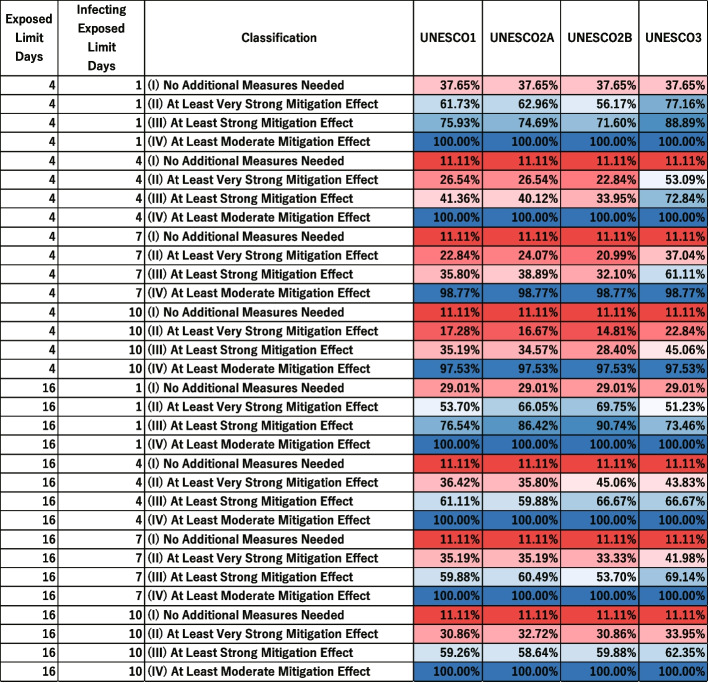
Classification of Intervention Effectiveness in the Early Stage (see Appendix B. for information about how to read the table)

Figures 8 and 9 show the distribution of scenario-specific mean $I_{\text {peak}}$ values for each parameter set, and the resulting effectiveness categories, under conditions where the exposed limit days were 4 or 16 and the infecting exposed limit days were 1, 4, 7, or 10. All results are shown in Appendix B, Figs. 18, 19, 20, and 21. For each combination of exposed limit days and infecting exposed limit days, there were 162 possible parameter combinations (see Table 3 in Appendix B). Figures 8 therefore plot the distributions of scenario-specific mean $I_{\text {peak}}$ values across 162 scenarios.

This corresponds to the early stages of an outbreak, where the period between exposure to the virus and symptom onset, and the time period during which an infected individual can transmit the virus, have both been identified.

Figure 8 compares the effectiveness of different interventions. It shows that, as previously noted by Takahashi [24], the UNESCO interventions do not always follow a strict order of superiority (i.e., UNESCO3, UNESCO2B, UNESCO2A, UNESCO1). For instance, when exposed limit days was 4 and infection exposed limit days was 1, UNESCO3 was more effective than the other interventions in Fig. 8. However, when exposed limit days was 16 and infection exposed limit days was 1, UNESCO2B was more effective.

Additionally, as infecting exposed limit days increased, the percentage of scenarios where an intervention was effective generally decreased. However, under two different conditions (when exposed limit days was set at 16 and infecting exposed limit days could be either 7 or 10), UNESCO2B was marginally more effective when infecting exposed limit days was 10. In Fig. 8, the boxplot for infecting exposed limit days of 10 was slightly lower than that for 7 days, indicating lower values of the scenario-specific mean $I_{\text {peak}}$. Additionally, the proportion in the “(III) At Least Strong Mitigation Effect” category of Fig. 8 was 53.70% when infecting exposed limit days was 7, but increased to 59.88% when it was 10, suggesting that the number of parameter scenarios in which the intervention was effective had increased.

These results show that effective intervention strategies cannot be determined simply by whether infecting exposed limit days and exposed limit days are long or short.

When exposed limit days was 4 or 16 and infecting exposed limit days was 1, the percentage of scenarios classified as “(I) No Additional Measures Needed” was around 30% in Fig. 9. This suggests that in some scenarios, intervention may not be necessary.

When exposed limit days was 4 and infecting exposed limit days ranged from 7 to 10, the percentage classified as “(IV) At Least Moderate Mitigation Effect” did not reach 100% in Fig. 9. This implies that no UNESCO intervention alone could reduce the peak infection count below the Strong Mitigation Threshold under those conditions. This scenario would therefore require additional measures.

### Middle stage

**Fig. 10:**
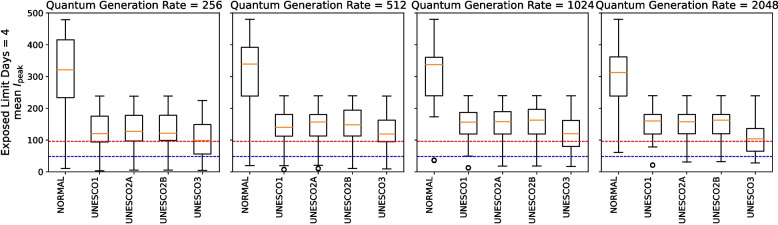
Effectiveness of UNESCO attendance strategies in the middle stage (exposed limit days = 4; quantum generation rate = 256, 512, 1024, and 2048). The blue dashed line represents the very strong mitigation threshold, defined as 10% of the total student population (48 students). The red dashed line represents the strong mitigation threshold, defined as 20% of the total student population (96 students)

**Fig. 11:**
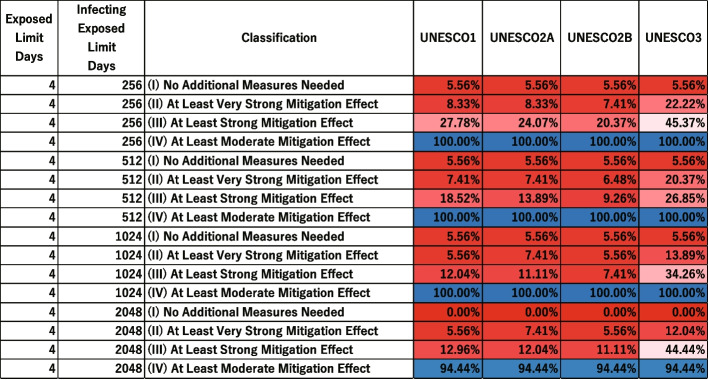
Classification of intervention effectiveness in the middle stage (see Appendix B for information about how to read the table)

Figures 10 and 11 show the distribution of scenario-specific mean $I_{\text {peak}}$ values for each parameter set, and the resulting effectiveness categories, when the exposed limit days was 4 and the quantum generation rate was 256, 512, 1024, or 2048. All results are shown in Appendix B, Figs. 22, 23, 24, and 25. For each combination of exposed limit days and quantum generation rate, there were 108 possible parameter combinations (see Table 3 in Appendix B). Figure 10 therefore plots the distributions of scenario-specific mean $I_{\text {peak}}$ values across 108 scenarios.

In general, as the quantum generation rate increased, the percentage of scenarios where the intervention was effective decreased in Appendix B: Figs. 22, 23, 24, and 25. However, the boxplot for UNESCO3 shows a higher distribution at a quantum generation rate of 512 than 256 or 1024 in Fig. 10. The lowest value for the “(III) At Least Strong Mitigation Effect” category of UNESCO3 with exposed limit days set to 4 appeared when the quantum generation rate was 512 (Fig. 11). The values for quantum generation rates of 256 and 1024 were higher. This suggests that, paradoxically, certain interventions may become more effective at a relatively high quantum generation rate.

Focusing on “(IV) At Least Moderate Mitigation Effect”, the value was consistently 100% when the quantum generation rate was low in Figs. 11 and 25 in Appendix B. However, when the quantum generation rate was high, the value dropped below 100% in some scenarios. This implies that it is possible to suppress the peak infection count below the Strong Mitigation Threshold as long as the quantum generation rate remains below a certain threshold.

### Late stage

**Fig. 12:**
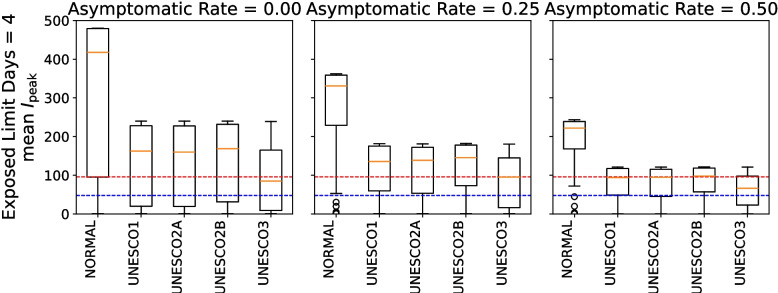
Effectiveness of UNESCO attendance strategies in the late stage (exposed limit days = 4; asymptomatic rate = 0.00, 0.25, and 0.50). The blue dashed line represents the very strong mitigation threshold, defined as 10% of the total student population (48 students). The red dashed line represents the strong mitigation threshold, defined as 20% of the total student population (96 students)

**Fig. 13:**
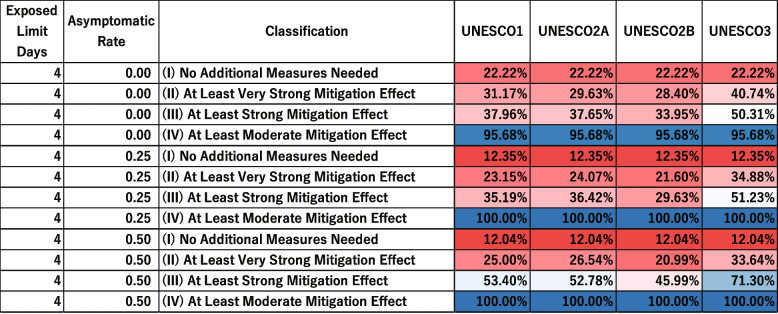
Classification of intervention effectiveness in the late stage (see Appendix B for information about how to read the table)

Figures 12 and 13 show the distribution of scenario-specific mean $I_{\text {peak}}$ values for each parameter set, and the resulting effectiveness categories, under conditions where the exposed limit days was 4 and the asymptomatic rate was 0.00, 0.25, or 0.50. All results are presented in Appendix B, Figs. 26 and 27. For each combination of exposed limit days and asymptomatic rate, there were 324 possible parameter combinations (see Table 3 in Appendix B). Figures 10 therefore plot the distributions of scenario-specific mean $I_{\text {peak}}$ values across 324 scenarios.

Focusing on UNESCO2A in Fig. 12, the proportion of scenarios where the box falls below the line for “(II) At Least Very Strong Mitigation Effect” (red dashed line) is smaller at an asymptomatic rate of 0.25 than at asymptomatic rates of 0.00 and 0.50. When focusing on the results of “(II) At Least Very Strong Mitigation Effect”, the direction of effectiveness based on the asymptomatic rate was not consistent, unlike the findings for infecting exposed limit days and quantum generation rate in Fig. 13. For example, in UNESCO2A, the effectiveness fluctuated from 29.63% at an asymptomatic rate of 0.00 to 24.07% at 0.25 and to 26.54% at 0.50.

As the asymptomatic rate increases, the number of asymptomatic infected individuals rises. This allows the disease to spread further through these individuals. However, asymptomatic individuals are not counted in the peak infection count, meaning that an increase in the asymptomatic rate does not necessarily lead to a higher peak infection count. This explains the observed fluctuations and inconsistencies.

Focusing on “(III) At Least Strong Mitigation Effect”, the percentage of effective intervention measures generally increased with the asymptomatic rate Figs. 26 and 27 in Appendix B.

### Applied simulation

This section describes the application of the proposed framework to two specific infectious diseases, to illustrate the decision-making process. The first infectious disease was COVID-19, where we used the characteristics that have been identified in previous studies. The second was a hypothetical infectious disease, designed to demonstrate the necessity of adaptive decision-making as characteristics become known in stages.

#### Simulated application to COVID-19

This subsection describes the simulated decision-making process for applying the proposed framework to COVID-19. The parameters for the disease were set as infected limit days = 16, infecting exposed limit days = 4, exposed limit days = 4, quantum generation rate = 64, and asymptomatic rate = 0.25.

**Fig. 14:**
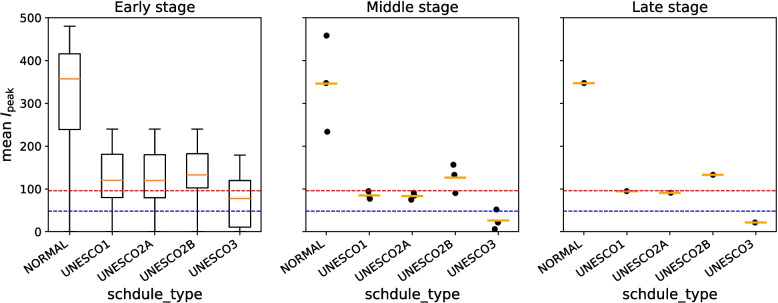
Simulation results of COVID-19 under the proposed framework (early, middle, and late stages). The blue dashed line represents the very strong mitigation threshold, defined as 10% of the total student population (48 students). The red dashed line represents the strong mitigation threshold, defined as 20% of the total student population (96 students). In the initial and middle stages, where the number of samples is five or fewer, only individual data points are plotted instead of boxplots

**Fig. 15:**
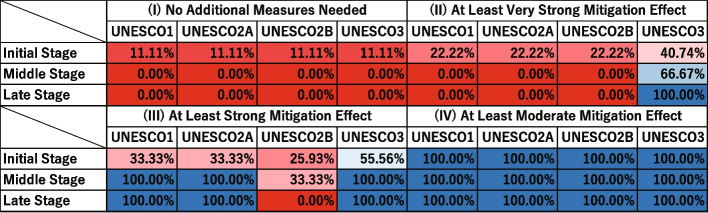
Classification of intervention effectiveness for COVID-19 (early, middle, and late stages) (see Appendix B for information about how to read the table)

Figure 14 illustrates the distribution of scenario-specific mean $I_{\text {peak}}$ for the initial stage, middle stage, and late stage. Figure 15 shows the proportions of categories for each scenario across these three stages. In the initial stage, the values of infected limit days = 16, infecting exposed limit days = 4, and exposed limit days = 4 were assumed to be known. The possible scenario patterns therefore consisted of combinations of quantum generation rate = 1, 32, 64, 128, 256, 512, 1024, 2048, and 4096 with asymptomatic rate = 0.00, 0.25, and 0.50, giving 27 combinations in total. The outcomes of these 27 scenarios were plotted. In the middle stage, the values of infected limit days = 16, infecting exposed limit days = 4, exposed limit days = 4, and quantum generation rate = 64 were assumed to be known. The possible scenario patterns were therefore limited to the three combinations of asymptomatic rate = 0.00, 0.25, and 0.50, and their results were plotted. In the late stage, all parameter values were assumed to be known, and the result of this single scenario was plotted.

First, we assumed that the infected limit days, infecting exposed limit days and exposed limit days would become known in the initial stage. The left panel of Fig. 14 represents the results for the initial stage. In this stage, the distribution of UNESCO3 showed the lowest results across the measures. The effectiveness of each UNESCO measure at this stage is shown in the “Initial Stage” row of Fig. 15. The “(I) No Additional Measures Needed” column in Fig. 15, shows that NORMAL conditions could keep the peak infection count below the Very Strong Mitigation Threshold in just 11.11% of scenarios. Next, UNESCO3 was the most effective in suppressing the peak infection count to the Very Strong Mitigation Threshold. The other three options were lower, and all similar. UNESCO3 remained the most effective in targeting the Strong Mitigation Threshold. UNESCO1 and UNESCO2A were about the same level of effectiveness, and UNESCO2B was the least effective. Finally, in the “(IV) At Least Moderate Mitigation Effect” category, all measures had a value of 100%, showing that all the UNESCO measures would provide a better infection control effect than NORMAL, regardless of the values of quantum generation rate or asymptomatic rate determined later.

On the basis of these results, selecting UNESCO3 was considered the most appropriate decision in the initial stage. However, there was still a considerable percentage of scenarios where even UNESCO3 failed to keep the peak infection count below the Strong Mitigation Threshold. Additional measures beyond school attendance policies should therefore also be considered under these circumstances.

Next, in the middle stage, the quantum generation rate emerges—in this scenario, 64. The middle panel of Fig. 14 represents the results for the middle stage. It shows that UNESCO3 fell below the Very Strong Mitigation Threshold in most scenarios. UNESCO1 and UNESCO2A fell below the Strong Mitigation Threshold in all scenarios. However, UNESCO2B exceeded the Strong Mitigation Threshold in most scenarios. The effectiveness of each UNESCO measure at this stage is shown in the “Middle Stage” row of Fig. 15. The “(I) No Additional Measures Needed” column in Fig. 15 shows that the NORMAL scenario could no longer keep the peak infection count below the Very Strong Mitigation Threshold in any scenario (0%). Only UNESCO3 remained effective for suppressing the peak infection count to the Very Strong Mitigation Threshold with a percentage of 66.67%. UNESCO1, UNESCO2A, and UNESCO2B showed 0%. This means that regardless of the asymptomatic rate, none of them could keep the peak infection count below the Very Strong Mitigation Threshold.

However, with respect to Strong Mitigation, UNESCO1, UNESCO2A, and UNESCO3 all showed values of 100%. This indicates that UNESCO1 and UNESCO2A would also be worth consideration if the goal was to keep the peak infection count below the Strong Mitigation Threshold, rather than the Very Strong Threshold. This suggests that, in situations where measures such as UNESCO3 are difficult to implement, UNESCO1 and UNESCO2A may serve as reasonable alternatives.

On the basis of these results, UNESCO3 was still the most appropriate choice in the middle stage. However, depending on the school environment and economic conditions, UNESCO1 and UNESCO2A could also be regarded as candidate options.

Finally, in the late stage, the asymptomatic rate was determined to be 0.25. The right panel of Fig. 14 represents the results for the late stage. In the late stage of Fig. 14, UNESCO3 fell below the Very Strong Mitigation Threshold in all scenarios. UNESCO1 and UNESCO2A fell below the Strong Mitigation Threshold in all scenarios. However, UNESCO2B exceeded the Strong Mitigation Threshold in all scenarios. This means that only UNESCO3 could suppress the peak infection count below the Very Strong Mitigation Threshold with 100% effectiveness. However, if the goal was strong mitigation, UNESCO1 and UNESCO2A also showed values of 100%, indicating that they would be worth consideration.

On the basis of these results, under these conditions, UNESCO3 was considered the most appropriate choice. However, as in the Middle Stage, UNESCO1 or UNESCO2A might also be suitable, depending on the school and economic context. If so, additional measures beyond school attendance schedules would be necessary.

#### Simulated application to a hypothetical infectious disease

This subsection discusses the scenario where the proposed framework was applied to a hypothetical infectious disease. The parameters for this hypothetical disease were set as infected limit days = 10, infecting exposed limit days = 1, exposed limit days = 16, quantum generation rate = 256, and asymptomatic rate = 0.25.

**Fig. 16:**
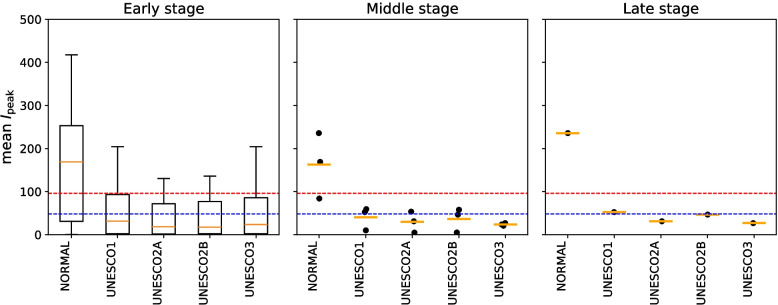
Simulation results of a hypothetical infectious disease under the proposed framework (early, middle, and late stages). The blue dashed line represents the Very Strong Mitigation Threshold, defined as 10% of the total student population (48 students). The red dashed line represents the strong mitigation threshold, defined as 20% of the total student population (96 students). In the initial and middle stages, where the number of samples was five or fewer, only individual data points were plotted instead of boxplots

**Fig. 17:**
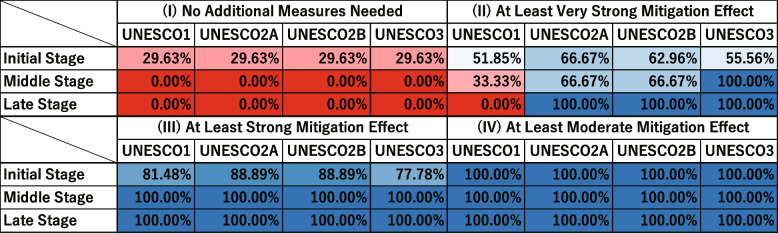
Classification of intervention effectiveness for a hypothetical infectious disease (early, middle, and late stages) (see Appendix B for information about how to read the table)

**Fig. 18:**
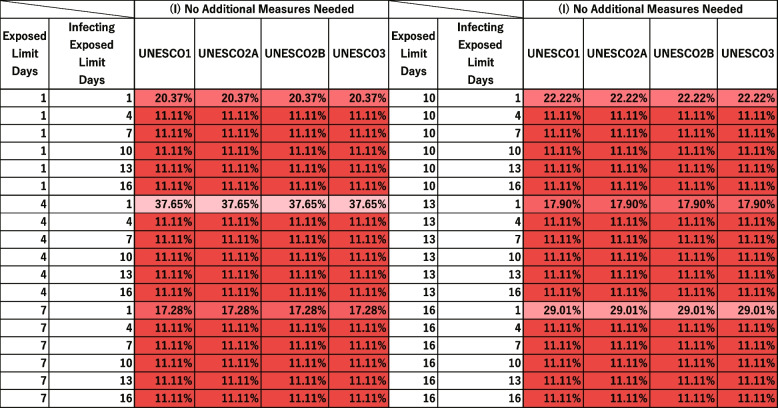
Early stage: exposed limit days and infecting exposed limit days: (I) no additional measures needed

**Fig. 19:**
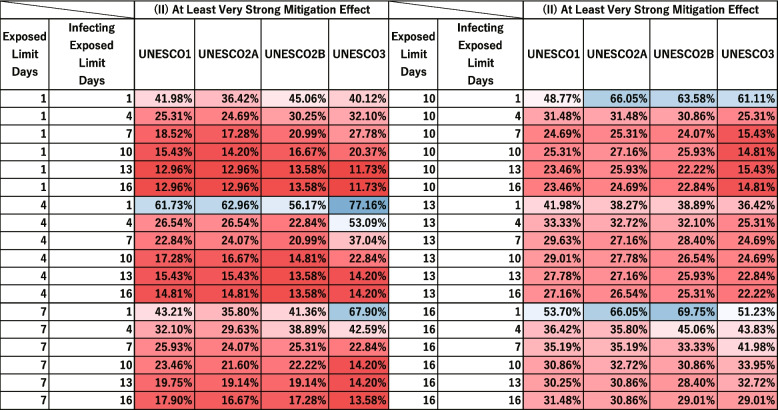
Early stage: exposed limit days and infecting exposed limit days: (II) at least very strong mitigation effect

**Fig. 20:**
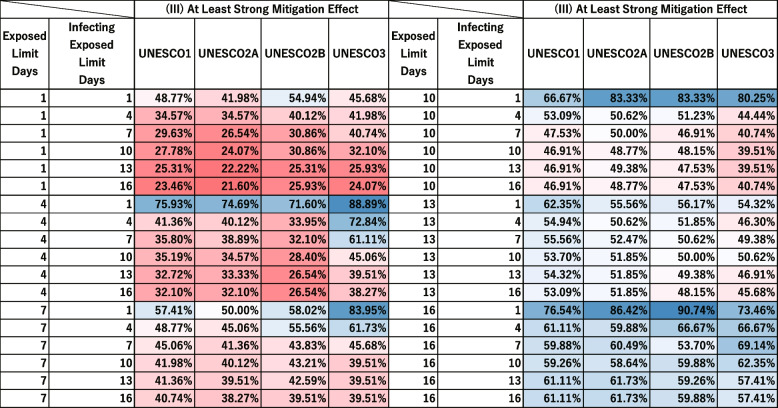
Early stage: exposed limit days and infecting exposed limit days: (III) at least strong mitigation effect

**Fig. 21:**
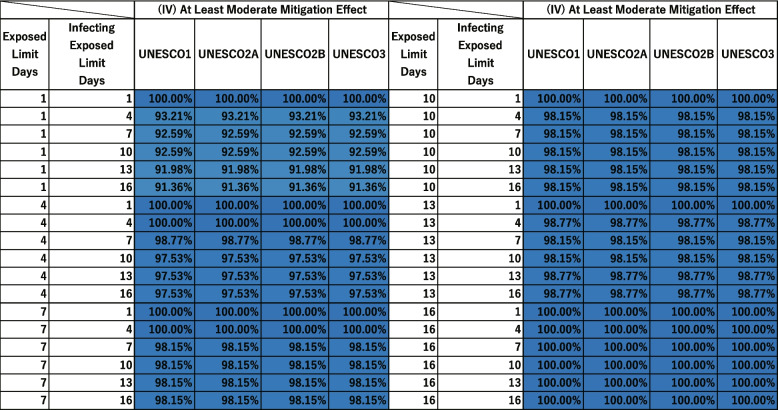
Early stage: exposed limit days and infecting exposed limit days: (IV) at least moderate mitigation effect

**Fig. 22:**
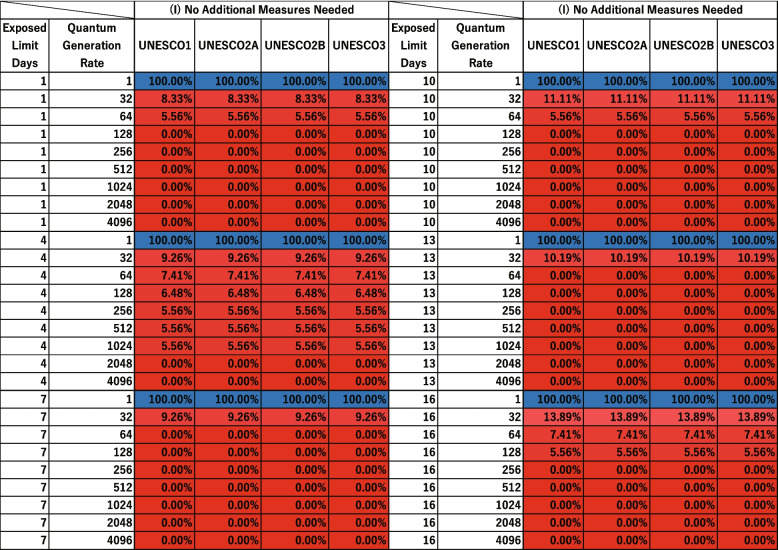
Middle stage: exposed limit days and quantum generation rate: (I) no additional measures needed

**Fig. 23:**
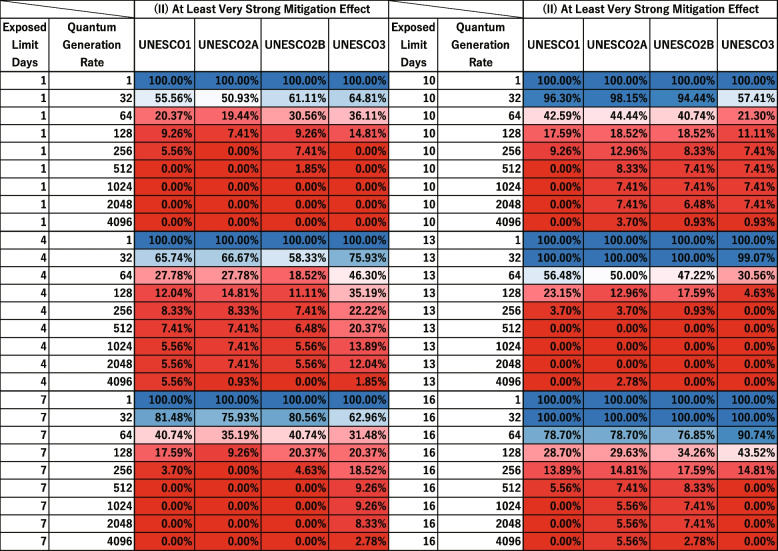
Middle stage: exposed limit days and quantum generation rate: (II) at least very strong mitigation effect

**Fig. 24:**
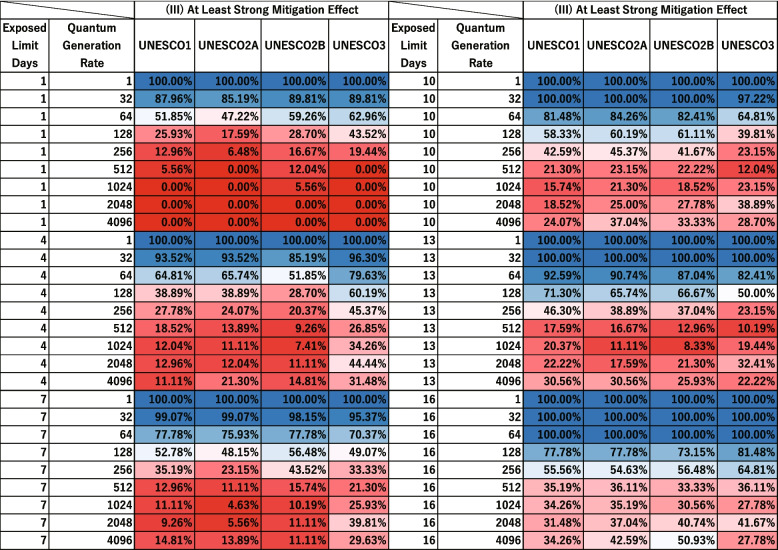
Middle stage: exposed limit days and quantum generation rate: (III) at least strong mitigation effect

**Fig. 25:**
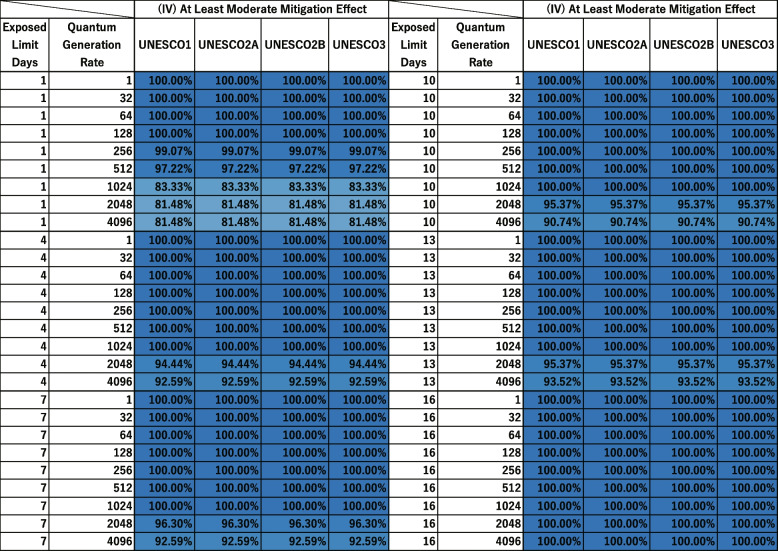
Middle stage: exposed limit days and quantum generation rate: (IV) at least moderate mitigation effect

**Fig. 26:**
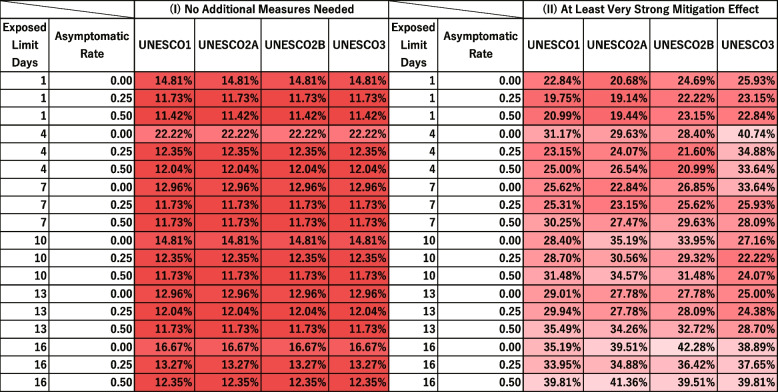
Late stage: exposed limit days and asymptomatic rate: (I) no additional measures needed and (II) at least very strong mitigation effect

**Fig. 27:**
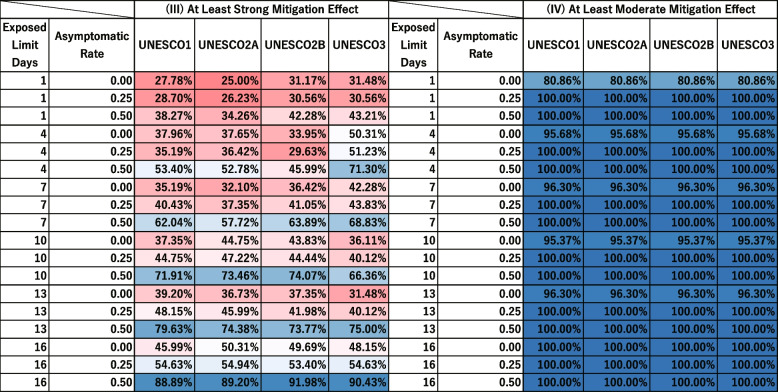
Late stage: exposed limit days and asymptomatic rate: (III) at least strong mitigation effect and (IV) at least moderate mitigation effect

Figure 16 illustrates the distribution of scenario-specific mean $I_{\text {peak}}$ for the initial stage, middle stage, and late stage. Figure 17 shows the proportions of categories for each scenario across these three stages. In the initial stage, the values of infected limit days = 10, infecting exposed limit days = 1, and exposed limit days = 16 were assumed to be known. The possible scenario patterns therefore consisted of combinations of quantum generation rate = 1, 32, 64, 128, 256, 512, 1024, 2048, and 4096 with asymptomatic rate = 0.00, 0.25, and 0.50, resulting in 27 combinations in total. The outcomes of these 27 scenarios were plotted. In the middle stage, the values of infected limit days = 10, infecting exposed limit days = 1, exposed limit days = 16, and quantum generation rate = 256 were assumed to be known. The possible scenario patterns were therefore limited to the three combinations of asymptomatic rate = 0.00, 0.25, and 0.50, and their results were plotted. In the late stage, all parameter values were assumed to be known, and the result of this single scenario was plotted.

First, we assumed that the infected limit days, infecting exposed limit days and exposed limit days would become known in the initial stage. The left panel of Fig. 16 represents the results for the initial stage. In this stage, the distribution of UNESCO2A gave the lowest results. The effectiveness of each UNESCO measure at this stage is shown in the “Initial Stage” row of Fig. 17. The “(I) No Additional Measures Needed” column in Fig. 17 shows that NORMAL measures could keep the peak infection count below the Very Strong Mitigation Threshold in 29.63% of scenarios. UNESCO2A was the most effective at suppressing the peak infection count to the Very Strong Mitigation Threshold, at 66.67%. It was followed by UNESCO2B (62.96%), UNESCO3 (55.56%), and UNESCO1 (51.85%). For measures targeting the Strong Mitigation Threshold, UNESCO2A and UNESCO2B were the most effective at 88.89%, followed by UNESCO1 at 81.48% and UNESCO3 at 77.78%. All measures reached a value of 100% in the “(IV) At Least Moderate Mitigation Effect” category, indicating that regardless of the values of quantum generation rate or asymptomatic rate, all UNESCO measures would provide better infection control than NORMAL.

Selecting UNESCO2A was therefore considered the most appropriate decision in the initial stage. UNESCO2B was similarly effective, and could also be a viable option, depending on factors such as school operations, learning outcomes, regional economy, and household conditions. The percentage of scenarios where the peak infection count was kept below the Strong Mitigation Threshold was high. There would therefore be no immediate need to consider additional mitigation measures beyond school attendance policies.

Next, in the middle stage, the quantum generation rate of 256 emerges. The middle panel of Fig. 16 represents the results for the middle stage. It shows that the NORMAL scenario could no longer keep the peak infection count below the Very Strong Mitigation Threshold in any scenario (0%). UNESCO3 was the most effective at suppressing the peak infection count to the Very Strong Mitigation Threshold, with a value of 100%. This means that regardless of the asymptomatic rate, UNESCO3 would successfully contain the peak infection count below the Very Strong Mitigation Threshold. UNESCO2A and UNESCO2B followed at 66.67%, with UNESCO1 at 33.33%. All measures were 100% effective at suppressing the Strong Mitigation Threshold, confirming their utility.

On the basis of these results, UNESCO3 was considered the most appropriate decision in the middle stage. There was almost no scenario where the peak infection count failed to be contained below the Strong Mitigation Threshold. There would therefore be no need to consider additional mitigation measures beyond school attendance policies.

Finally, in the late stage, the asymptomatic rate is determined to be 0.25. The right panel of Fig. 16 shows the results for the late stage. This shows that UNESCO2A, UNESCO2B, and UNESCO3 were all equally effective in keeping the peak infection count below the Very Strong Mitigation Threshold, at 100%. However, UNESCO1 was entirely ineffective, with a 0% value. However, when considering suppression to the Strong Mitigation Threshold, UNESCO1 also achieved a 100% effectiveness rate.

On the basis of these results, selecting any of UNESCO2A, UNESCO2B, or UNESCO3 would be appropriate in the late stage. Each measure has different impacts on school operations, learning outcomes, regional economy, and household conditions. The most suitable measure should therefore be selected on the basis of these factors. There were also almost no scenarios where the peak infection count was not contained below the Strong Mitigation Threshold. There would therefore be no need to consider additional mitigation measures beyond school attendance policies.

## Discussion

In this study, we evaluated the effectiveness of various interventions for infectious diseases and validated the necessity and utility of our proposed framework. This section discusses the findings from the evaluations.

### The comprehensive evaluation

The comprehensive analysis confirmed that appropriate interventions vary with the stage of disease progression. The effectiveness of measures changes significantly with different disease characteristics such as exposed limit days, infecting exposed limit days, quantum generation rate, and asymptomatic rate.

In the initial stage, exposed limit days and infecting exposed limit days had a significant impact on the effectiveness of interventions. However, the effectiveness of measures could not be consistently determined solely on the basis of whether these values were long or short. Instead, it was necessary to select interventions to reflect specific conditions. For example, UNESCO3 tended to be effective when exposed limit days was short. As the exposed limit days increased, UNESCO2B became more effective in some scenarios. These results indicate that a uniform application of interventions is not sufficient, and an adaptive approach based on disease characteristics is required.

In the middle stage, the effectiveness of interventions was significantly influenced by quantum generation rate. When this was low, additional interventions were often unnecessary. As the quantum generation rate increased, the effectiveness of interventions generally declined. However, in some scenarios, certain ranges of quantum generation rate actually enhanced the effectiveness of measures, suggesting that the dynamics of infection are influenced by nonlinear factors.

In the late stage, the impact of asymptomatic rate became apparent. This altered the proportion of asymptomatic carriers, leading to inconsistent effects of interventions. However, higher asymptomatic rates sometimes improved the effectiveness of interventions aiming to deliver a Strong Mitigation Effect. These results indicate that the presence of asymptomatic carriers complicates intervention effectiveness, necessitating further detailed analysis.

### Effectiveness of the proposed framework

In the simulation for COVID-19, during the initial stage, only infected limit days, infecting exposed limit days, and exposed limit days were known. At this stage, UNESCO3 was identified as the most effective measure. However, a certain percentage of scenarios could not be contained below the Strong Mitigation Threshold, suggesting the need for additional interventions beyond school attendance policies.

In the middle stage, when the quantum generation rate became known, NORMAL measures could not keep the peak infection count below the Very Strong Mitigation Threshold. UNESCO3 provided a certain level of containment, but other interventions struggled to achieve adequate suppression. It was therefore deemed appropriate to continue using UNESCO3, while preparing to implement additional interventions.

In the late stage, when the asymptomatic rate was determined, UNESCO3 successfully contained the peak infection count below the Very Strong Mitigation Threshold in all scenarios. UNESCO1 and UNESCO2A contained the peak below the Strong Mitigation Threshold, while UNESCO2B exceeded this threshold in all scenarios. This indicated that only UNESCO3 could suppress the peak infection count below the Very Strong Mitigation Threshold with full effectiveness.

These results demonstrate that as the characteristics of an infectious disease become gradually known, the selection of appropriate interventions changes. In our simulations, UNESCO3 was identified as sufficient to manage COVID-19 effectively. However, in the real world, additional interventions such as lockdowns and vaccination campaigns were implemented. This discrepancy highlights the limitations of simulation-based approaches, which cannot fully capture the complexity of real-world decision-making and societal factors.

To place these findings in the broader context of real-world COVID-19 countermeasures, it is useful to note that multiple complementary interventions were widely implemented in practice. Combining online and in-person learning was reported to maintain educational continuity while mitigating infection risk [51]. Lockdowns and mandatory social distancing measures were also introduced globally to maintain infection counts at sufficiently low levels [52]. This is also consistent with our findings. The widespread vaccination campaigns helped mitigate the impact of the asymptomatic rate, ultimately contributing to infection control.

These results indicate that while the simulation findings are broadly consistent with real-world COVID-19 countermeasures, the discrepancy created by the introduction of additional interventions in practice highlights the limitations of simulation. Therefore, the proposed framework suggests potential utility as a supplementary tool for real-world decision-making.

In the simulation for a hypothetical infectious disease, during the initial stage, there were a relatively high percentage of scenarios where the peak infection count remained below the Very Strong Mitigation Threshold even with NORMAL school attendance. However, UNESCO2A and UNESCO2B were the most effective interventions. Selecting UNESCO2A in the initial stage was therefore deemed appropriate, with UNESCO2B as a viable alternative.

In the middle stage, once the quantum generation rate was determined, NORMAL attendance was no longer able to keep the peak infection count below the Very Strong Mitigation Threshold. However, UNESCO3 successfully contained the peak infection count below this threshold in 100% of scenarios. Thus, in the middle stage, selecting UNESCO3 was the most appropriate course of action.

In the late stage, when the asymptomatic rate was determined, UNESCO2A, UNESCO2B, and UNESCO3 all achieved 100% effectiveness in containing the peak infection count below the Very Strong Mitigation Threshold. By contrast, UNESCO1 was ineffective at that level, although it could contain the peak infection count below the Strong Mitigation Threshold. In the late stage, any of UNESCO2A, UNESCO2B, and UNESCO3 were therefore appropriate, with the decision being based on the impact on school operations, learning outcomes, regional economy, and household conditions.

These findings reaffirm that as an infectious disease’s characteristics become progressively known, the selection of appropriate interventions changes. The middle stage played a crucial role in decision-making because the selection of interventions at this stage had a significant impact on infection control outcomes.

The results highlight that using the proposed framework for specific infectious diseases can support well-informed intervention choices while also considering school operations, learning outcomes, regional economy, and household conditions.

### Comparison with related studies

The dynamic decision-making framework proposed in this study selects school attendance strategies by the stage of the outbreak of an infectious disease. It is consistent with the findings of previous modeling research, but also offers a novel contribution. Since the onset of the COVID-19 pandemic, several simulation studies have evaluated the effects of non-pharmaceutical interventions in school settings.

Many modeling studies emphasize the importance of combining multiple interventions rather than relying on a single measure. For example, Kucharski et al. [53] demonstrated that testing, isolation, contact tracing, and physical distancing were most effective when implemented together. In school-specific contexts, Colosi et al. [54] showed that regular screening of students substantially reduced transmission in French schools, while Borges et al. [12] highlighted that the scale of outbreaks in Brazil strongly depended on both community incidence and the effectiveness of testing and tracing systems. These results are consistent with our finding that staggered attendance policies alone are insufficient and must be supplemented with additional interventions.

Examining specific forms of staggered attendance, Rozhnova et al. [55] analyzed Dutch schools and found that rotation-based attendance strategies could effectively reduce transmission without full closures. Similarly, rotation-based modeling in the Czech Republic indicated that weekly alternating schedules and half-class weekly rotations were comparably effective [56]. This aligns with our result that UNESCO3 (weekly cohort rotation) is particularly effective in the early stage of an outbreak or when infectiousness is high.

Most of these related studies were conducted specifically in the context of COVID-19 and relied on fixed parameter values, often tied to particular variants or outbreak conditions. By contrast, our framework explicitly incorporates the temporal progression of pandemics, where key epidemiological parameters—such as latent period, transmissibility, and the proportion of asymptomatic cases—become known only gradually. This approach demonstrates how the optimal school attendance strategy may shift across different stages of an outbreak, making the framework more broadly applicable not only to COVID-19 but also to future emerging infectious diseases.

When comparing these results with the actual COVID-19 containment measures implemented globally, our findings align with the hybrid learning models widely adopted in many countries, where online and in-person classes were combined to maintain educational continuity while reducing infection risk. In addition, staggered or rotation-based school attendance policies—such as alternating days or weekly cohort rotations—were implemented in Europe and Asia as non-pharmaceutical interventions. These real-world practices are consistent with our result that UNESCO3 (weekly cohort rotation) is particularly effective. Furthermore, lockdowns and mandatory social distancing were introduced globally to suppress transmission, and widespread vaccination campaigns later mitigated the impact of asymptomatic carriers.

Taken together, these comparisons demonstrate both the practical relevance and the flexibility of our proposed framework. In this study, we employed the Takahashi school infection simulation model (SVISM) as the basis for our analysis [24]. However, the proposed decision-making framework is not restricted to this specific model. The underlying structure can be adapted to alternative modeling approaches or modified according to the characteristics of the target environment to be reproduced. This flexibility allows the framework to be applied beyond the present case, making it suitable for evaluating diverse settings such as workplaces, public facilities, or other educational systems.

## Conclusion

In this study, we proposed a framework for dynamically adjusting infection control measures in schools, reflecting the stepwise process of identifying characteristics of emerging infectious diseases. We found that infection control measures can be examined while taking into account both educational and social impacts. The effectiveness of infection control can be improved by selecting appropriate staggered school attendance methods at each stage of disease identification (initial, middle, and late).

The key findings of this study can be summarized as follows:

- *The Need for Dynamic Adaptation*: As the characteristics of an infectious disease become clearer, the optimal staggered school attendance policy changes. For example, UNESCO3 (weekly rotation attendance) is often the most effective strategy in the initial stage. However, once the infectivity level is determined, UNESCO2B or UNESCO2A may become more appropriate choices because they are equally effective but less disruptive to learning.
- *Balancing Infection Control and Educational Continuity*: Staggered school attendance helps suppress infection, but the continuity of education and its impact on families and society must also be considered. A balance between infection risk and education can be achieved by setting appropriate thresholds.
- *The Influence of Infectivity and Asymptomatic Rate on Policy Selection*: When the infectivity level (quantum generation rate) is high, long-term staggered attendance (e.g., UNESCO3) is effective. However, when the asymptomatic rate is high, enhanced ventilation and the integration of online learning become essential.

### Policy implications

The findings of this study provide important insights for policymakers in designing infection control measures in school environments.

- Implementing a dynamic plan that allows flexible adjustment of interventions as disease characteristics are identified can support both education continuity and infection control.
- Instead of applying uniform measures, it is advisable to select policies based on specific school environments, infection risks, and other contextual factors.

### Future challenges

This study has several limitations, and further investigation is needed to address the following research challenges:

- *Developing Models That Consider Changes in Social Behavior*: This study did not explicitly consider changes in social behavior, such as mask-wearing rates among students or vaccination coverage. Future research should incorporate these factors into simulation models.
- *Accounting for Regional Differences*: Education environments and healthcare infrastructure vary by region. Optimizing infection control measures for specific regions is necessary, particularly in developing countries where online learning may be less accessible because of limited internet infrastructure.
- *Evaluating Long-Term Impacts*: This study focused on short-term infection control. However, the long-term effects, such as lost educational opportunities and students’ mental health, require further evaluation.
- *Application to Other Environments*: This framework was designed primarily for school environments, but it could be extended to workplaces and public facilities. Future research should explore its applicability in diverse settings.

### Conclusion

This study proposed a framework for dynamically adapting infection control measures in school environments, as the characteristics of emerging infectious diseases become progressively known. The proposed method supports both infection suppression and educational continuity, and can be applied to infectious diseases with different characteristics.

When disease characteristics are unclear, our findings suggest that adaptive policy selection is essential. The proposed framework could be extended beyond schools to workplaces, public facilities, and other environments, to support infection control planning.

In future, researchers should apply this framework to a broader range of environments and identify optimal measures under different regional and contextual conditions. However, our findings are expected to contribute to decision-making in future pandemic responses.

This study is subject to several model-specific assumptions that may limit the direct applicability of its findings. The model assumes a fixed school size of 600 students, a constant class size of 30, and a simulation time horizon of 120 days. It also assumes homogeneous mixing within classrooms and fixed seating arrangements. These simplifications do not capture the diversity of contact patterns, class size variations, or dynamic changes in attendance, and therefore caution should be exercised when extrapolating the results to schools with different configurations or more complex social contact structures.

In addition to these structural assumptions, the scope of our analysis was restricted to infection dynamics. The disadvantages listed in Section 2.3.3 were not explicitly modeled in this study. Our approach focused on infection dynamics, and future work should extend the framework to incorporate additional metrics, such as educational loss, operational burden, and other socio-economic impacts, to evaluate the trade-offs between epidemiological benefits and associated disadvantages.

## Data Availability

No datasets were generated or analysed during the current study.

## Appendix A: Clarifying the characteristics of infectious diseases

The characteristics of infectious diseases are revealed progressively over time.

### SARS

The characteristics of SARS-CoV-1 became known in the order of incubation period, infectiousness, and transmission route. SARS emerged in China in November 2002, coming to international attention after spreading to Hong Kong in March 2003. In April 2003, Peiris et al. [57] used case data from Hong Kong to show that the median incubation period of SARS was 4–6 days, with a maximum of approximately 10 days. The following month, Donnelly et al. [58] analyzed the epidemiological determinants of SARS-CoV-1 transmission in Hong Kong and also reported that the median incubation period ranged from 4 to 6 days. This finding provided the basis for guidelines recommending an isolation period of over 10 days, contributing to measures to prevent the spread of infection.

Estimates of the basic reproduction number (R0), representing the infectiousness of SARS, were promptly sought as the outbreak progressed. In May 2003, Lipsitch et al. [59] estimated the R0 of SARS to be in the range of 2–3 based on early data, indicating high transmissibility. In June 2003, Riley et al. [60] analyzed infection clusters in Hong Kong and estimated the R0 to range from 1.7 to 3.6. They also discussed the impact of public health interventions. These insights underscored the necessity of swift and stringent measures to contain the spread of SARS.

### COVID-19

The characteristics of COVID-19 emerged in the order of incubation period, infectiousness, and the proportion of asymptomatic cases. The timeline for the emergence of COVID-19 characteristics can be inferred from WHO reports [61] and academic papers.

The incubation period of COVID-19 was initially mentioned in the WHO situation report on January 26, 2020 [62]. It was subsequently estimated to be **2–10 days** in the report of January 27 [63]. Further investigations led to a revision in the report published on February 19, 2020 [64]. This estimated the median incubation period to be 5–6 days, with a range of 0–14 days [64]. This finding, based on the time from exposure to symptom onset, provided the scientific basis for many countries to set a 14-day isolation period.

The basic reproduction number (R0) of COVID-19 was first estimated in the report of March 6, 2020, which indicated an R0 of 2–2.5 [65]. This highlighted the importance of social distancing and contact tracing to prevent the spread of infection.

Asymptomatic cases were first noted in WHO reports from January 2020, though transmission risk was initially considered very rare [66, 67]. The February 1, 2020 report identified seven asymptomatic cases outside China, while the February 27, 2020 report acknowledged increasing asymptomatic case reports and emphasized the need for serological testing [67, 68]. By March 2020, WHO introduced protocols to estimate the asymptomatic fraction of infections [69]. A June 2020 narrative review by Oran and Topol suggested that approximately 40–45% of SARS-CoV-2 infections may be asymptomatic, supporting recommendations for expanded testing programs to include individuals without symptoms [20].

### Summary

In the early stages of emerging infectious diseases, the incubation period and infectious period are revealed through contact tracing and secondary infection analysis [14–16]. In the middle stages, measures of infectiousness, such as the basic reproduction number, are assessed through animal experiments and statistical investigations to determine the ease of spread [17, 18]. Finally, in the later stages, the proportion of asymptomatic cases and their potential for transmission are clarified through population and antibody testing [19–23].

## Appendix B: The visualization of results

Here, each cell in Figs. 18–27 represents the proportion of simulation runs within a given parameter set that were classified into each effectiveness category. By aggregating these proportions across the full factorial design, the visualizations provide an overarching view of the relative effectiveness of different interventions. In particular, they highlight which school attendance strategies remain robust across a wide range of plausible epidemiological conditions, rather than focusing on single-case outcomes.

Table 3 summarizes the calculation method used to generate the visualizations of results. For each stage (early, middle, and late), simulations were conducted for a set of parameter combinations, and the outcomes of each intervention measure were classified into five categories: (I) No Additional Measures Needed, (II) Very Strong Mitigation Effect, (III) Strong Mitigation Effect, (IV) Moderate Mitigation Effect, and (V) No Significant Effect.

The Number of Scenarios rows indicate how many parameter combinations fall into each category. The Cumulative Number of Scenarios rows show the total number of scenarios when categories are accumulated in order from (I) to (IV). The Cumulative Percentage of Scenarios rows represent these cumulative totals as percentages of the total number of parameter combinations for that stage.

To facilitate comparisons among intervention measures, the proportions were accumulated from “(I) No Additional Measures Needed” up to“(IV) Moderate Mitigation Effect”, and then labeled as “at least” that level. For example, the percentage of scenarios classified as “(III) At Least Strong Mitigation Effect” includes all scenarios classified as “(I) No Additional Measures Needed”, “(II) Very Strong Mitigation Effect”, or “(III) Strong Mitigation Effect”.

By structuring the results in this manner, it is possible to visualize the percentage of parameter settings for each stage in which certain interventions are highly effective versus those where additional measures yield little benefit. This approach enables the identification of intervention strategies that are robust across a wide range of infectious disease characteristics, providing a view of the relative effectiveness of various policies.

**Table 3:** Calculation method for visualizing intervention effectiveness

Parameters	Early stage	Middle stage	Late stage
Exposed Limit Days	1 (in the top left-hand cell)	1 (in the top left-hand cell)	1 (in the top left-hand cell)
Infecting Exposed Limit Days	1 (in the top left-hand cell)	1, 4, 7, 10, 13, 16	1, 4, 7, 10, 13, 16
Infected Limit Days	4, 7, 10, 13, 16, 19	4, 7, 10, 13, 16, 19	4, 7, 10, 13, 16, 19
Quantum Generation Rate	1, 32, 64, 128, 256, 512, 1024, 2048, 4096	1 (in the top left-hand cell)	1, 32, 64, 128, 256, 512, 1024, 2048, 4096
Asymptomatic Rate	0.00, 0.25, 0.50	0.00, 0.25, 0.50	0.00 (in the top left-hand cell)
Possible Parameter Combinations	162	108	324
**Number of Scenarios**			
(I) No Additional Measures Needed	33	162	19
(II) Very Strong Mitigation Effect	35	0	18
(III) Strong Mitigation Effect	11	0	8
(IV) Moderate Mitigation Effect	83	0	85
**Cumulative Number of Scenarios**			
(I) No Additional Measures Needed	33	162	19
(II) At Least Very Strong Mitigation Effect	68	162	37
(III) At Least Strong Mitigation Effect	79	162	45
(IV) At Least Moderate Mitigation Effect	162	162	130
**Cumulative Percentage of Scenarios**			
(I) No Additional Measures Needed	20.37%	100.00%	11.73%
(II) At Least Very Strong Mitigation Effect	41.98%	100.00%	22.84%
(III) At Least Strong Mitigation Effect	48.77%	100.00%	27.78%
(IV) At Least Moderate Mitigation Effect	100.00%	100.00%	80.86%

## Code availability

The code for the simulation model is available at the link below https://github.com/satoshi-takahashi-lab/school-virus-infection-simulator.
